# Rare scrotal chylous effusion: A case report

**DOI:** 10.1177/2050313X241231368

**Published:** 2024-02-08

**Authors:** Amy Ducharme, Benoît Côté

**Affiliations:** 1 Division of Dermatology, Department of Medicine, Université de Montréal, Canada; 2 Department of Dermatology clinic, Centre hospitalier de l’Université de Montréal, QC﻿, Canada

**Keywords:** Dermatology, Klippel-Trenaunay syndrome (KTS), scrotal chylous effusion

## Abstract

Klippel–Trenaunay syndrome is a rare congenital malformation predominantly affecting lower limb. In most cases, it is characterized by a classic triad of cutaneous capillary malformation (port-wine stain), lymphatic and venous abnormalities, in association with variable soft tissue and bone overgrowths. We describe a 48-year-old male presenting on the genitalia several whitish vesicles discharging a milky fluid compatible with chyle. Extensive radiology workup revealed pelvic megalymphatic malformations. Pelvic lymphatic ligations and bleomycin sclerotherapy only allowed a partial improvement. Given the high potential of recurrence, the patient will soon undergo a genetic evaluation for PIK3CA gene mutation and may need further systemic treatment with Sirolimus. As this scrotal chylous effusion in the setting of Klippel–Trenaunay syndrome is rare and highly affects the quality of life, we wanted to raise awareness of this entity and its management.

## Introduction

Described for the first time in the early 20th century, the Klippel–Trenaunay syndrome (KTS) is a rare congenital disorder characterized by a classic triad of cutaneous capillary malformation (“port-wine stain”), lymphatic and venous abnormalities in association with variable overgrowth of soft tissue and bone.^1^ KTS predominantly affects the lower limb, although it can occur less commonly in the upper extremity and trunk. Lymphatic malformations can manifest internally or externally in various locations, leading to a range of clinical complications.^2^ To our knowledge, no instances of scrotal involvement in the setting of KTS have been reported in the literature. We present a case of a 48-year-old male with a previous history of KTS associated with scrotal chylous effusion. Specific investigation is discussed.

## Case report

**Figure 1.**
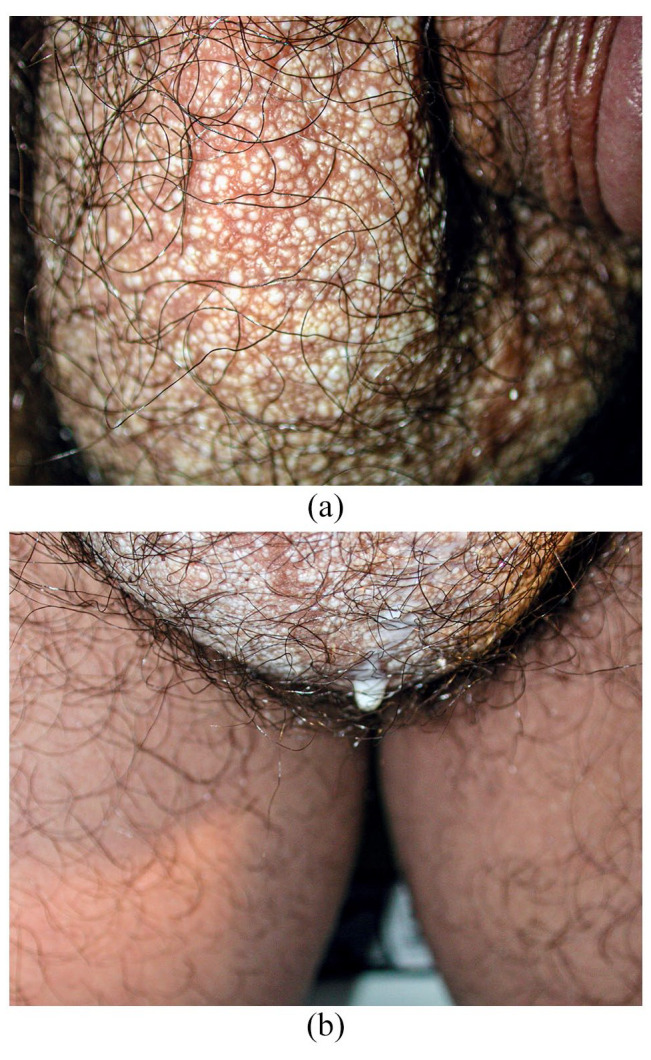
(a) Multiple whitish millimetric vesiculopapules and (b) milky fluid-filled channels on scrotum. Note: Color of papules ranges from creamy white to translucent.

**Figure 2.**
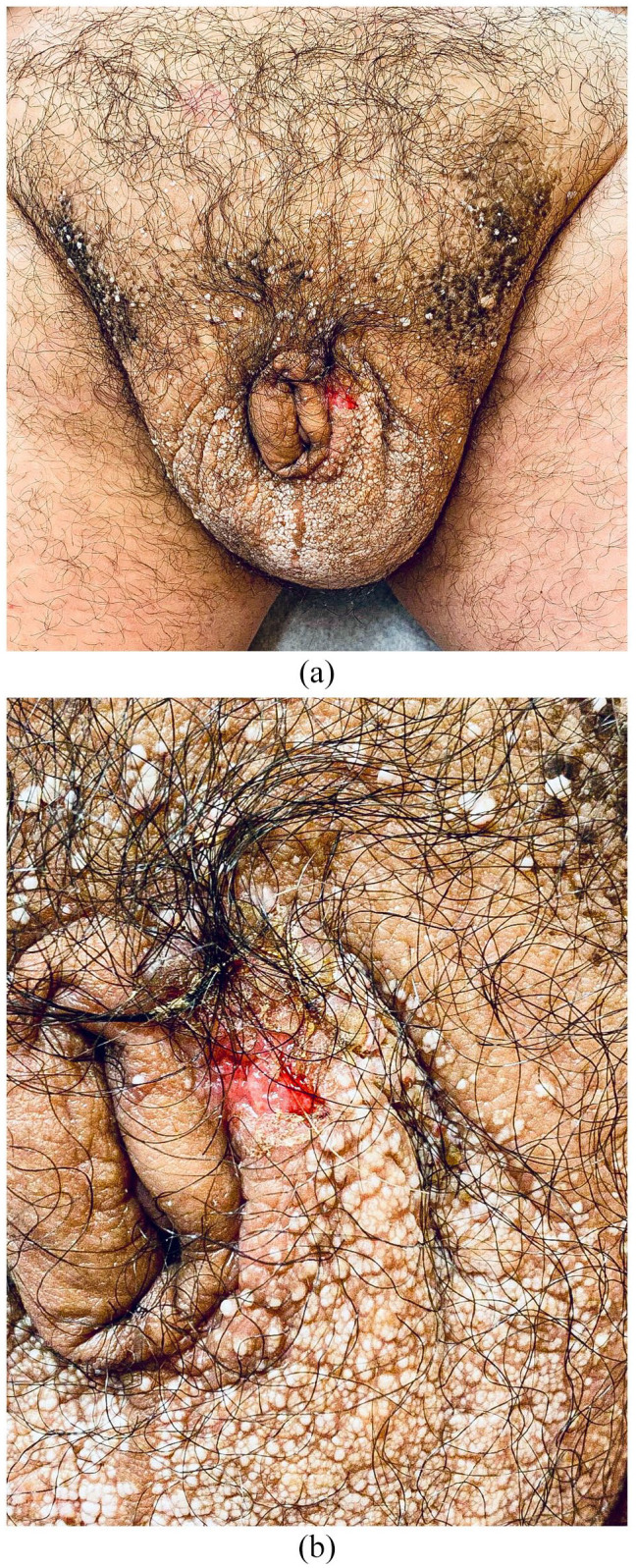
(a) Severe scrotal oedema with penile invagination and (b) Bowen’s disease presenting as an ulcer on the left scrotal area.

A 48-year-old male patient presented with a recurrent milky scrotal discharge with several tiny milia-like lesions (Figure 1a). Past medical history includes KTS with a congenital malformation of his left lower limb. Scrotal discharge started during adolescence at the age of 15 and progressively worsened. He was 30 years old when he first consulted in dermatology in 2001. Following physical exertion, a white milky fluid spontaneously leaked from the scrotal area (Figure 1b). Only prolonged dorsal decubitus position could relieve the patient. Biochemical analysis of the scrotal fluid was compatible with chyle, with triglyceride levels measuring 43.32 mmol/L, and lipoprotein analysis confirming the presence of chylomicrons. An MRI (magnetic resonance imaging) also revealed megalymphatics in the pelvis. In 2003, the patient underwent pelvic lymphatic ligations followed by sclerotherapy in 2004. These specialized procedures were performed in Boston, where the expertise was available. Following these surgeries, the patient experienced approximately 90% improvement in his condition for about a decade. However, the scrotal chylous effusion gradually recurred in 2014. In 2021, the patient returned to our clinic with the same initial symptoms. The impact on his quality of life was major: he had to wear protective pads daily and could not do any physical activity as it was exacerbating the scrotal flow. Physical examination revealed severe scrotal edema with penile invagination (Figure 2a). Several whitish millimetric vesiculopapules with punctiform lymphangiectasias were observed throughout the scrotal area. Despite wearing compression stockings, persistent edema was observed in the left lower limb. Additionally, a superficial ulcer was identified on the left side of the scrotum (Figure 2b). An excisional biopsy was performed, confirming a diagnosis of squamous cell carcinoma in situ (Bowen’s disease). The patient underwent standard excision by a urologist, but subsequently developed a local skin infection with delayed wound healing. He was managed with intravenous and oral antibiotics. In summary, the findings were consistent with a diagnosis of recurrent scrotal chylous effusion secondary to pelvic megalymphatics in the context of KTS. In 2022, the patient was referred to angioradiology department at our hospital, where he recently underwent sclerotherapy with local bleomycin injection. Currently, the patient is being followed by a multidisciplinary team.

## Discussion

Our manuscript presents a rare case of recurrent scrotal chylous effusion secondary to pelvic megalymphatic malformations in the setting of KTS. Although this localization for chylous effusion is uncommon, analysis of the fluid oozing from the patient’s scrotum confirmed the presence of chyle. Our patient underwent an extensive radiologic workup that confirmed the abnormalities responsible for his condition. Given the rarity of this type of lymphatic malformation, no published guidelines exist for managing this condition. Some authors have previously reported similar scrotal presentations, but they were unrelated to KTS.^3,4^ Suehiro et al.^5^ reported a case of a 25-year-old man with cutaneous scrotal chylous effusion secondary to primary lymphoedema who responded well to lymphaticovenous anastomoses. However, pelvic lymphatic ligations and bleomycin sclerotherapy performed under angioradiological guidance only resulted in partial improvement in our patient. Therefore, additional sclerotherapy may be necessary. Furthermore, the patient will soon undergo a genetic evaluation for PIK3CA gene mutation.^6^ If needed, systemic treatment with Sirolimus could be considered.^7^ Given the well-known risk of neoplastic complications in chronic lymphedema, clinical surveillance is warranted. Moreover, episodic cellulitis is a common complication, likely attributed to poor lymphatic drainage in the affected area.^8^ Considering the complexity of management, KTS patients with scrotal chylous effusion should receive multidisciplinary medical care regarding the physical, psychological, and social aspects of the disease. In conclusion, we describe scrotal chylous effusion as a rare complication resulting from pelvic megalymphatics in the setting of KTS. Due to these abnormalities, the risk of recurrence is deemed to remain high even after ligations and bleomycin sclerotherapy. Thus, symptomatic or cosmetically worrisome lesions warrant clinical follow-up by a dermatologist in addition to multidisciplinary care management. As this scrotal chylous effusion is clinically challenging and significantly impacts the patient's quality of life, we wanted to raise awareness of this entity and its management.
